# The role of tumour suppressor PDCD4 in beta cell death in hypoxia

**DOI:** 10.1371/journal.pone.0181235

**Published:** 2017-07-27

**Authors:** Sandeep Kumar, Claire E. Marriott, Nouf F. Alhasawi, Adrian J. Bone, Wendy M. Macfarlane

**Affiliations:** Diabetes Research Group, School of Pharmacy and Biomolecular Sciences, University of Brighton, Brighton, United Kingdom; University of South Alabama Mitchell Cancer Institute, UNITED STATES

## Abstract

**Objective:**

Hypoxia is known to induce pancreatic beta cell dysfunction and apoptosis. Changes in Programmed Cell Death Gene 4 (PDCD4) expression have previously been linked with beta cell neogenesis and function. Our aim was to investigate the effects of hypoxia on cell viability, PDCD4 expression and subcellular localisation.

**Methods:**

MIN6 beta cells and ARIP ductal cells were exposed to 1% (hypoxia) or 21% O_2_ (normoxia) for 12 or 24 hours. MTT assay, HPI staining, scanning electron microscopy, western blotting and immunocytochemistry analyses were performed to determine the effect of hypoxia on cell viability, morphology and PDCD4 expression.

**Results:**

24 hour exposure to hypoxia resulted in ~70% loss of beta cell viability (P<0.001) compared to normoxia. Both HPI staining and SEM analysis demonstrated beta cell apoptosis and necrosis after 12 hours exposure to hypoxia. ARIP cells also displayed hypoxia-induced apoptosis and altered surface morphology after 24 hours, but no significant growth difference (p>0.05) was observed between hypoxic and normoxic conditions. Significantly higher expression of PDCD4 was observed in both beta cells (P<0.001) and ductal (P<0.01) cells under hypoxic conditions compared to controls. PDCD4 expression was localised to the cytoplasm of both beta cells and ductal cells, with no observed effects of hypoxia, normoxia or serum free conditions on intracellular shuttling of PDCD4.

**Conclusion:**

These findings indicate that hypoxia-induced expression of PDCD4 is associated with increased beta cell death and suggests that PDCD4 may be an important factor in regulating beta cell survival during hypoxic stress.

## Introduction

Hypoxia can occur in many pathological conditions and is defined as an oxygen level ≤ 2%. Ambient air is 21% oxygen; however, most mammalian tissues exist at 2%-9% oxygen [1]. Cellular oxygen tension depends on a balance between oxygen supply and demand, with an imbalance leading to hypoxia [1, 2]. There have been recent reports on the effect of hypoxia on pancreatic islets, inducing a reduction in beta cell survival post transplantation, associated with the low oxygenation of grafted pancreatic islets [3] and resulting in higher numbers of islets being required to restore glucose homeostasis [4]. It is clear that high vascular density and oxygenation of transplanted islets is necessary in order to prevent beta cell dysfunction and apoptosis by hypoxia [5–7].

Beta cell death by apoptosis [8] contributes significantly to both Type 1 Diabetes (T1D) and Type 2 Diabetes (T2D) [9, 10]; however, the molecular mechanisms behind this are poorly understood. It has recently been suggested that PDCD4 (programmed cell death protein 4) and NFκB (nuclear factor kappa B) form a unique regulatory axis that controls programmed cell death of beta cells [11]. Also, our previous data have shown that PDCD4 plays a crucial role in beta cell neogenesis and function [12].

Programmed cell death 4 gene (*Pdcd4*) (also known as *MA-3*, *TIS*, *H731* and *DUG*) was first identified during an investigation into genes up-regulated in the process of apoptosis [13–16]. *Pdcd4* has recently shown to be a novel tumour suppressor gene demonstrating down-regulation or loss of expression in several types of cancer [17–25]. PDCD4 is ubiquitously expressed in normal tissue and up-regulation of the PDCD4 protein has been identified in both apoptotic as well as healthy cells [13]. Manipulation of PDCD4 expression by ectopically over-expressing [26] or knocking down expression [27, 28] have shown significant effects on cell growth and survival. How PDCD4 exerts its effect is not known; however, it has been reported that in the cytoplasm PDCD4 protein interacts with eukaryotic translation initiation factor 4A (eIF4A) via its MA-3 domain and inhibits its helicase activity, thus inhibiting cap-dependent translation [29, 30]. In addition, in the nucleus PDCD4 inhibits transcription factor activator protein-1 (AP-1) activity and controls gene transcription. Thus cellular localisation of PDCD4 is a potential regulator of its activity but conflicting data exist about the subcellular localisation of PDCD4. Some studies have found that PDCD4 is localized in the nucleus in normal cells and in the cytoplasm in cancer cells [19,31] whilst others have reported opposite findings [18]. This conflicting data might be due to the shuttling of PDCD4 between the cytoplasm and nucleus [32], cell cycle association of PDCD4 localisation [33] or cell type specific localisation of PDCD4 protein [34]. Subcellular localisation and expression of PDCD4 protein in pancreatic cells has not, however, been extensively studied to date.

The aim of this study was to investigate the expression and subcellular localisation of PDCD4 in MIN6 cells, a mouse pancreatic beta cell line and ARIP, a rat pancreatic ductal cell line, under both normoxic and hypoxic conditions. We demonstrate here for the first time that PDCD4 expression is increased under hypoxic conditions, as beta cell viability constantly decreases. Finally, we show that an increase in cytoplasmic PDCD4 expression is associated with apoptosis/necrosis in pancreatic beta cells exposed to hypoxia, suggesting that hypoxic conditions and elevated PDCD4 expression may contribute to beta cell loss in grafted islets post transplantation. In addition, our findings indicate that PDCD4 is localised to the cytoplasm of pancreatic cells and neither hypoxic nor serum starvation conditions are capable of inducing PDCD4 shuttling.

## Research design and methods

### Materials

DMEM low glucose (Cat No E15-806), DMEM-F12K (Cat No E15-012), foetal calf serum (FCS)(Cat No A15-104) and trypsin (Cat No L11-659) were purchased from PAA Laboratories, UK.

Antibodies to Programmed Cell Death Gene 4 (PDCD4) (Cat No ab51495), β-actin (Cat No ab8226), Lamin B1 (Cat No ab8226), HIF1-alpha (Cat No ab51608) and FITC conjugated anti-rabbit secondary antibodies (Cat No ab6717) were purchased from AbCam, UK. HRP-conjugated anti-rabbit (Cat No A0545), anti-mouse secondary antibodies (Cat No A5278), goat serum (Cat No G9023) and all laboratory chemicals and reagents were purchased from Sigma, UK unless otherwise stated.

### Cell culture

The mouse pancreatic β-cell line (Min6) [35] was used between passages 28 to 34 and cultured in Dulbecco’s Modified Eagle’s Medium (DMEM) with 5mM glucose containing 2mM L-glutamine and supplemented with 10% (V/V) FCS and 100 units/ml penicillin and streptomycin 100μg/ml. The rat pancreatic ductal cell line ARIP (ATCC number: CRL-1674) [36] was used between passages 18 to 26 and cultured in F-12K medium supplemented with 10% (V/V) FCS, 2mM L-glutamine, 100units/ml penicillin and streptomycin 100μg/ml. In all experiments cells were serum starved overnight in medium without FCS (to synchronise cells to G0). The following day cells were stimulated with standard culture medium containing 10% FCS and subsequently were exposed to normoxia (21% O_2_, 5% CO_2_) or hypoxia (1% O_2_, 5% CO_2_) at 37°C in a hypoxic chamber (COY Laboratory, US).

### MTT assay

MIN6 cells were seeded at 4×10^5^ cells/well and ARIP cells were seeded at 3×10^5^ cells/well in six well plates and incubated overnight at 5% CO_2_, at 37°C. Following specific experimental conditions, media was removed and cells were washed with phosphate buffered saline (PBS). 1ml of 0.5mg/ml MTT (3-(4, 5-dimethyl-2-thiazol)-2, 5-diphenyl-2H-tetrazolium bromide) (Cat No M5655) solution was added to each well and incubated for 1 hour. The MTT solution was then aspirated and 1ml DMSO (dimethyl sulfoxide) (Cat No 472301) solution added to each well for 5 minutes to dissolve the crystals. A 200μl sample from each well was transferred to a 96-well plate and absorbance was read at 540 nm using Multiscan Plate Reader (Thermo Fisher Scientific, UK). A DMSO alone sample was also analysed to provide a background control reading.

### Hoechst propidium iodide (HPI) staining

MIN6 cells were seeded at 4×10^5^ cells/well and ARIP cells were seeded at 3×10^5^ cells/well in six well plates and incubated overnight under standard culture conditions. Following specific experimental conditions, media was removed and cells were washed with PBS. HPI staining mixture was prepared in culture medium by mixing Hoechst (5mg/ml)/ Propidium iodide (1mg/ml) and 200μl was added to each well. Hoechst (Cat No B1155), propidium iodide (Cat No P4864). Cells were analysed by fluorescence microscopy (Zeiss, Axiovert 25) and images were captured at 10X magnification.

### Western blotting

MIN6 (4×10^5^ cells/ml) and ARIP (3×10^5^ cells/ml) cells were seeded at in T75 flask and incubated overnight under standard culture conditions. Following specific experimental conditions, media was removed and cells were washed with PBS. Nuclear and cytoplasmic extracts were prepared as follows: Cells were scraped from the base of the T75 flask and pelleted by centrifugation for 5 minutes. The supernatant was removed and the pellet re-suspended in 200μl buffer A (10mM HEPES pH 7.9; 10mM KCl, 0.1mM EDTA pH 8.0, EGTA pH 8.0, 1mM DTT, 1 X protease inhibitor cocktail in water) (Protease inhibitor cocktail, Pierce, Thermo Fisher Scientific, UK, Cat No 88266) and incubated on ice for 15 minutes. For cytoplasmic extracts: 12.5μl of 10% NP-40 added to buffer A and solution vortexed for 30 seconds. Cells were then centrifuged for 45 seconds. Supernatant was removed and snap frozen in liquid nitrogen. For nuclear extracts: Following cytoplasmic extraction, the pellet was re-suspended in 50μl buffer C (20mM HEPES pH 7.9; 400mM NaCl, 1mM EDTA pH 8.0, 1mM EGTA pH 8.0, 1mM DTT, 5% glycerol, 1 X protease inhibitor cocktail in water) and incubated with shaking for 1 hour at 4°C. Cells were centrifuged for 30 seconds and supernatant was removed and snap frozen in liquid nitrogen. Protein concentration was determined using Bio-Rad protein Assay Kit reagent (Biorad, Hemel Hempstead, UK) (Cat No 500–0006). 10μg of protein extracts were mixed (1:1 ratio) with SDS sample buffer (20% SDS; 0.1% of bromophenol blue; 1.25M sucrose; 1M Tris-HCl pH 6.8; 10% β-Mercaptoethanol), resolved at 10% SDS-PAGE and subsequently transferred to a nitrocellulose membrane (Amersham Biosciences, UK) (Cat No 28906837). Membranes were blocked using Tris-buffered saline with tween 20 (TBST) containing 10% skimmed dried milk and subsequently probed using Anti-PDCD4 (1:1000), Anti-β-actin (1:2000) and Anti-Lamin B1 (1:2000) antibodies. After washing, the membrane was incubated with HRP-conjugated secondary anti-rabbit or anti-mouse antibody and developed using an ECL-plus (enhanced chemiuminescence) detection kit (Pierce, Thermo Fisher Scientific, UK) (Cat No 80196). Densitometry analyses were performed using the Image J analysis system (ImageJ.nih.gov).

### Immunocytochemistry (ICC)

Subcellular localisation of PDCD4 was assessed by immunocytochemical staining. MIN6 cells were seeded at 4×10^5^ cells/well and ARIP cells were seeded at 3×10^5^ cells/well on sterile cover slips in six well plates and incubated overnight under standard culture conditions. Following specific experimental conditions, media was removed and cells were washed with PBS and fixed in 3.7% formalin in PBS for 10 minutes. Cells were then permeabilised with 0.1% triton X-100 in PBS followed by blocking in blocking buffer (10% Goat serum; 2% BSA; 0.2% Tween 20; 0.7% Glycerol in PBS) for 1 hour. Cells were incubated overnight with Anti-PDCD4 antibody (1:100) and FITC conjugated secondary anti-rabbit antibody (1:80) was applied. Coverslips with stained cells were mounted on glass slides using mounting medium containing DAPI (Vector laboratories, UK). Cells were finally analysed by confocal microscopy (Leica TCS SP5, Leica Microsystems, UK) and images were captured under oil immersion at 63X magnification.

### Statistics and densitometry

The data are expressed as mean ± SEM of at least three independent experiments. Statistical analyses were carried out the using 2-way ANOVA test and significance was determined at p-values of <0.05 (*), <0.01(**) and <0.001(***).

## Results and discussion

### Effect of hypoxia on pancreatic cell proliferation

**Fig 1:**
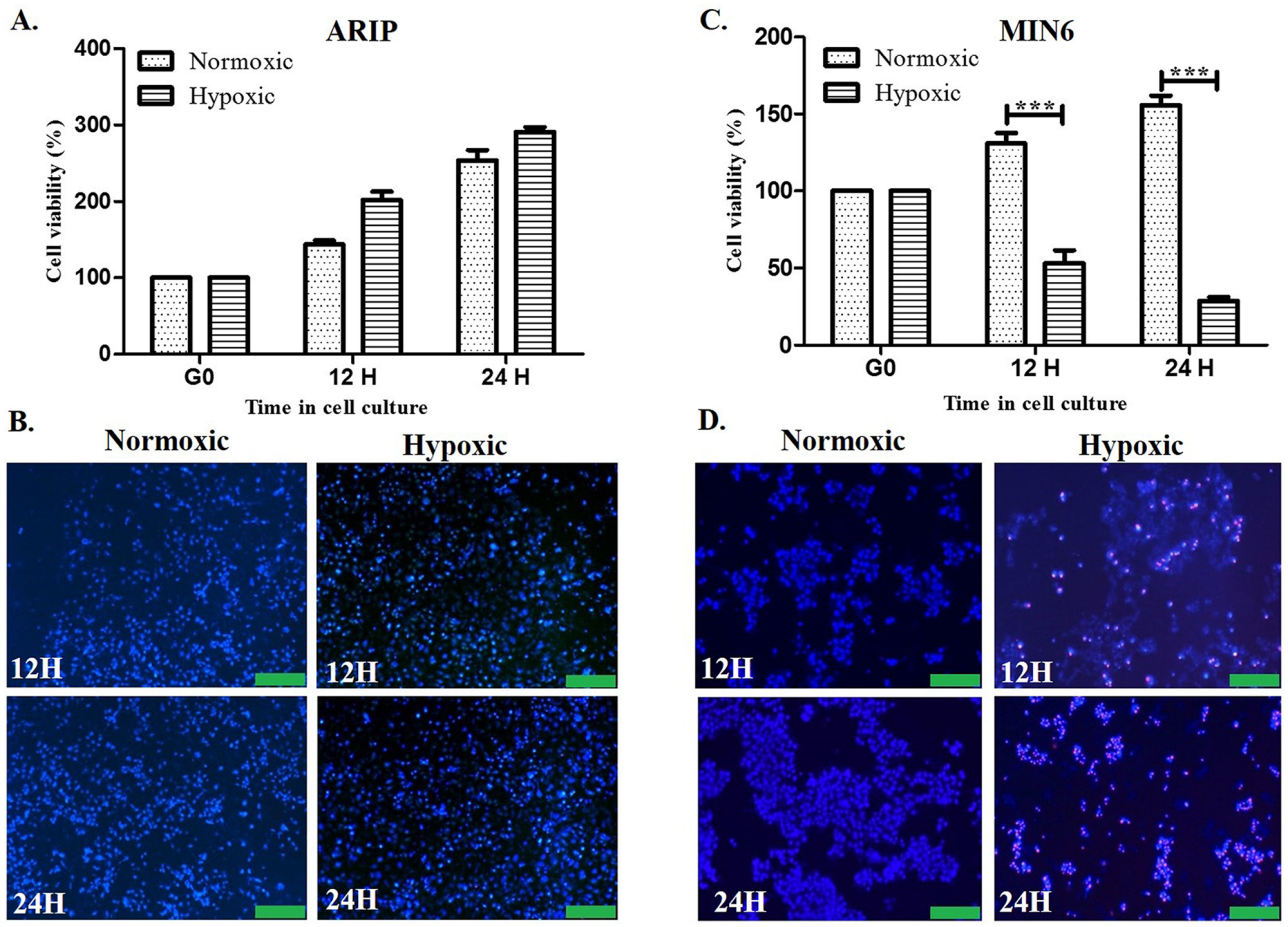
Effect of hypoxia on cell viability of ARIP and MIN6 cells analysed by MTT assay and HPI staining. (A) ARIP cells were exposed to hypoxia and no significant difference in viability was observed between hypoxic and normoxic conditions at 12 or 24 hour time points (p>0.05), error bar values represent mean +/- standard error. (B) Bright blue nuclei were observed indicating apoptosis triggered by hypoxia in ARIP cells. (C) In MIN6 cells, significant differences in viability between hypoxic and normoxic conditions at 12 hour (*** p<0.001) as well as 24 hour (*** p<0.001) time points were observed; error bar values represent mean +/- standard error. (D) Bright blue and red coloured nuclei were observed indicating apoptosis and necrosis triggered by hypoxia in MIN6 cells. (Scale bars = 300 μm).

To evaluate the effect of hypoxia on pancreatic cell proliferation, ARIP and MIN6 cells were exposed to hypoxia for 12 or 24 hours. After treatment, cell viability was determined by MTT assay and HPI staining. The viability of ARIP cells assessed by MTT assay showed that there was no significant difference in cell viability between hypoxic and normoxic conditions at all time points (p>0.05) (Fig 1(A)). However, assessment of viability of ARIP cells by HPI staining revealed that hypoxia induced apoptosis with substantial numbers of apoptotic cells demonstrating bright blue nuclear staining (Fig 1(B)). Exposure of β-cells to hypoxia revealed not only a detrimental effect on the viability of MIN6 cells but also significant growth difference between hypoxic and normoxic conditions at 12 hour (p<0.001) as well as 24 hour (p<0.001) time points (Fig 1(C)). HPI staining indicated that hypoxia induced both apoptosis and necrosis in MIN6 cells with both bright blue/red nucleus observed (Fig 1(D)). Overall, results indicate that hypoxia has a detrimental effect on the viability of pancreatic β-cells and induces apoptosis in pancreatic cells. These results were additionally confirmed by scanning electron microscopy analysis (S1 Fig).

### Effect of hypoxia on PDCD4 expression

**Fig 2:**
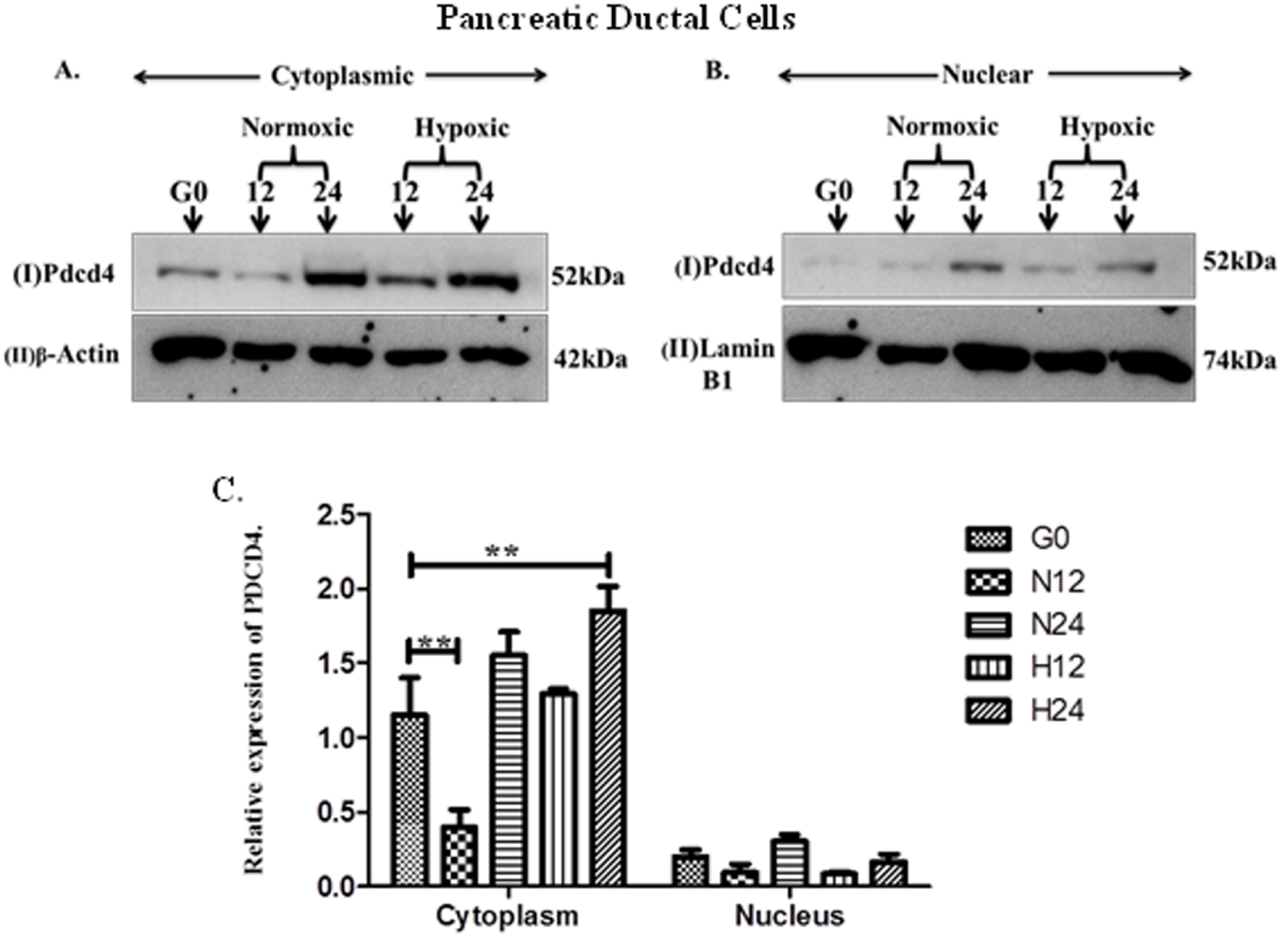
Hypoxia induced the expression of PDCD4 in ARIP cells. ARIP cells were cultured under serum starvation conditions (G0), normoxic or hypoxic conditions for 12 or 24 hours as indicated. Cytoplasmic and nuclear proteins were prepared and analysed by western blotting using an antibody specific to PDCD4. Panel A (I) represents PDCD4 (52kDa) protein expression in the cytoplasm (II) represents protein loading control β-actin (42kDa). Panel B (I) represents PDCD4 (52kDa) protein expression in the nucleus (II) represents protein loading control lamin B1 (74kDa). Panel C illustrates densitometry analyses, showing cytoplasmic PDCD4 relative to β-actin and nuclear PDCD4 relative to lamin B1. These results were reproduced in at least three separate experiments. Error bar values represent mean +/- standard error. **p<0.01 comparing the samples indicated.

**Fig 3:**
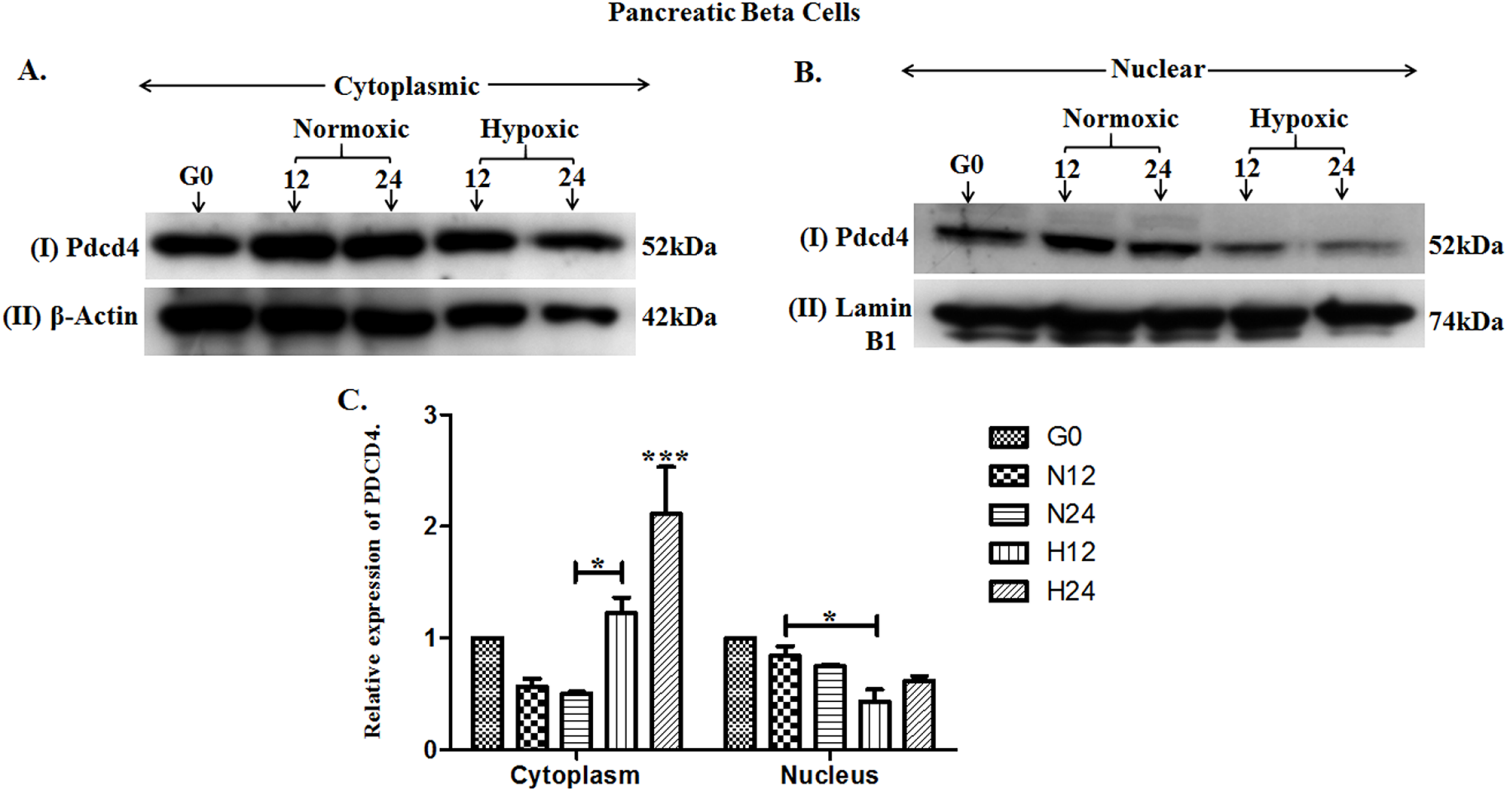
Hypoxia induced the expression of PDCD4 in MIN6 beta cells. MIN6 cells were cultured under serum starvation conditions (G0), normoxic and hypoxic conditions for 12 or 24 hours. Cytoplasmic and nuclear proteins were prepared and analysed by western blotting using an antibody specific to PDCD4. Panel A (I) represents PDCD4 (52kDa) protein expression in the cytoplasm (II) represents protein loading control β-actin (42kDa). Panel B (I) Represents PDCD4 (52kDa) protein expression in the nucleus (II) represents protein loading control lamin B1 (74kDa). Panel C illustrates densitometry analysis, showing cytoplasmic PDCD4 relative to β-actin and nuclear PDCD4 relative to lamin B1. These results were reproduced in at least three separate experiments. Error bar values represent mean +/- standard error. ***p<0.001, *p<0.05 comparing the sample indicated.

Our previous studies have identified the upregulation of *Pdcd4* both during the process of *in vivo* islet neogenesis and *in vitro* with PDCD4 expression observed in both beta cell and ductal cell lines [12]. We have therefore further examined the effect of hypoxia on PDCD4 expression. Western blotting (WB) was performed to investigate the expression of PDCD4 in ARIP and MIN6 cells in response to hypoxia and normoxia. Nuclear and cytoplasmic protein lysates of ARIP cells were subjected to WB and showed that PDCD4 was highly expressed in the cytoplasm compared to the nucleus of the cell (Fig 2). Densitometry analysis indicated that hypoxia induced expression of PDCD4 in the cytoplasm. Expression of PDCD4 was significantly higher in hypoxic conditions after 24 hours (p<0.01) and significantly lower in normoxic conditions after 12 hours (p<0.01) compared to basal level. WB results in MIN6 cells confirmed that hypoxia induced the expression of PDCD4 and that it was highly expressed in the cytoplasm compared to the nucleus of the cell (Fig 3). Densitometry analysis of MIN6 cells confirmed that expression of PDCD4 was significantly higher in hypoxic conditions after 24 hours (p<0.001) compared with all time points. In addition, densitometry also showed that there was no significant difference in expression of PDCD4 in ARIP cells under hypoxic and normoxic conditions (p>0.05). However, PDCD4 expression in MIN6 cells maintained under hypoxic conditions was significantly higher (p<0.001) compared to cells in normoxic conditions. Overall these results indicate that the increased expression of PDCD4 observed in MIN6 cells in hypoxic conditions may be a factor leading to β-cell death demonstrated in the viability studies.

### Cellular localisation of PDCD4 in pancreatic cells

**Fig 4:**
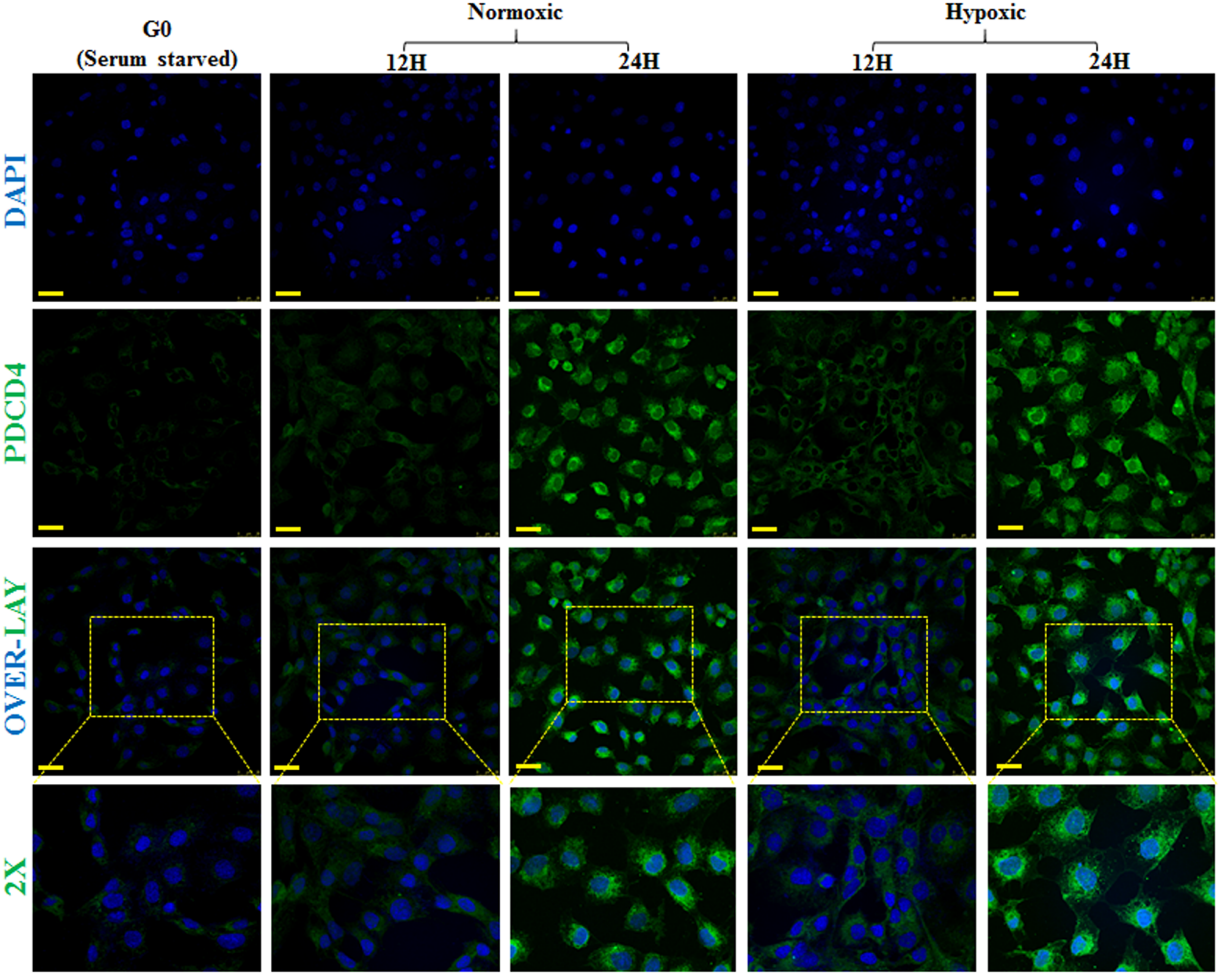
Sub-cellular localisation and expression of PDCD4 in ARIP ductal cells. Immunocytochemistry was performed using a specific antibody to PDCD4 and FITC labelled secondary antibody was used. Coverslips with cells were mounted on glass slides with mounting medium containing DAPI (Blue) which stains the nucleus of the cells. Cells were analysed by confocal microscopy and images were captured at 63X magnification. Results are representative of three separate experiments and images were representative of six separate fields. PDCD4 localized and expressing At G0: Cytoplasmic and very low expression; At N12: Cytoplasmic and low expression; At N24: very high cytoplasmic and low nuclear expression; At H12: Cytoplasmic expression and At H24 very high cytoplasmic and low nuclear expression. Over-all PDCD4 was highly expressed in the cytoplasm, with very low expression under serum starved conditions. (Scale bars = 25 μm).

**Fig 5:**
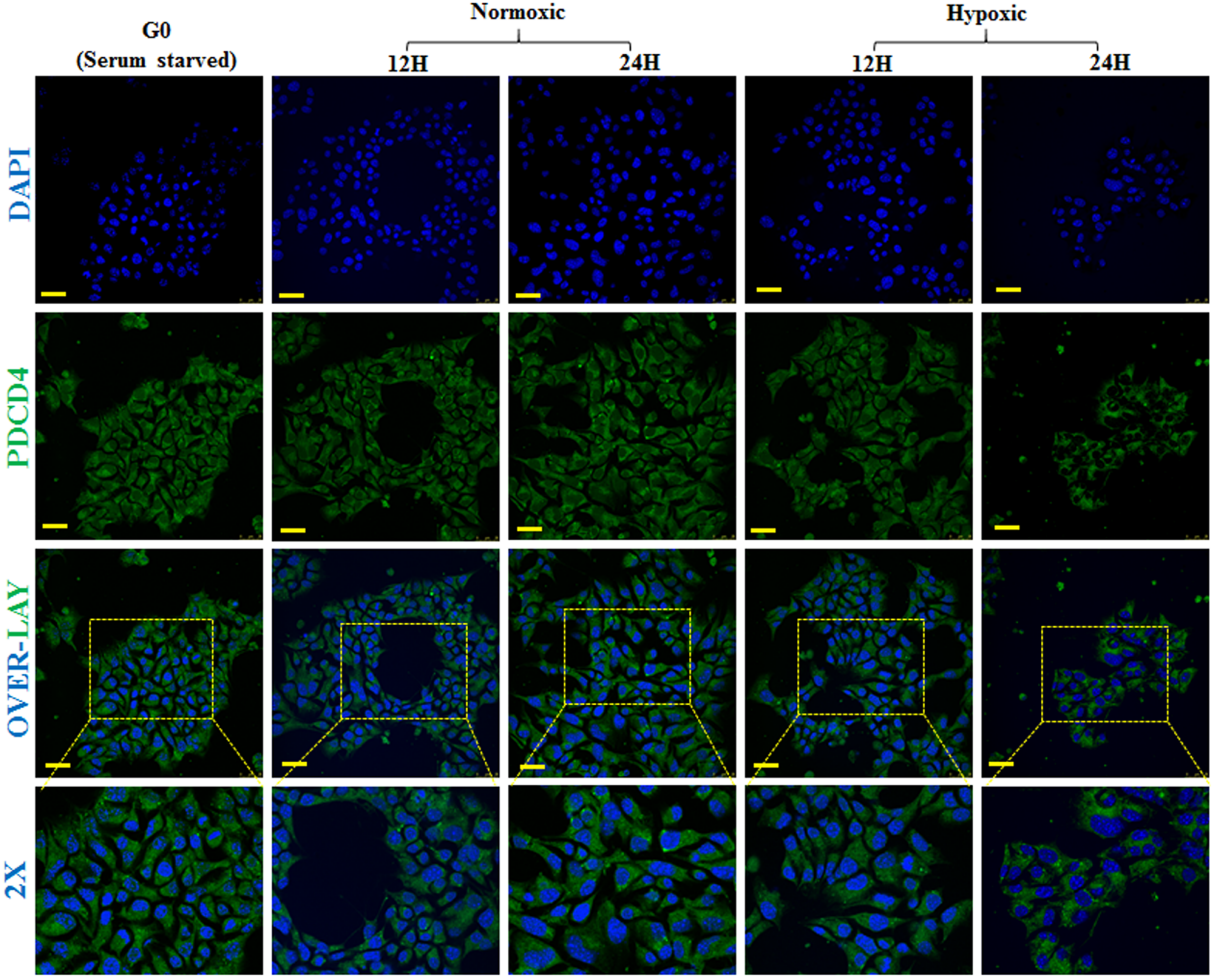
Sub-cellular localisation and expression of PDCD4 in MIN6 beta cells. MIN6 cells were grown on glass cover slips in six well plates and fixed at various time points i.e. at G0 (serum starvation), N12 & N24 (normoxic) and H12 & H24 (hypoxic). Immunocytochemistry was performed using a specific antibody to PDCD4 and FITC labelled secondary antibody was used. Coverslips with cells were mounted on glass slides with mounting medium containing DAPI (Blue) which stains the nucleus of the cells. Cells were analysed by confocal microscopy and images were captured at 63X magnification. Results are representative of three separate experiments and images were representative of six separate fields. PDCD4 localized and expressed at G0, N12, N24 and H12: Cytoplasmic and very low nuclear expression; At H24: High cytoplasmic expression. Overall, PDCD4 was highly expressed in the cytoplasm, with very low nuclear expression. (Scale bars = 25 μm).

There is conflicting data on the subcellular localisation of PDCD4 expression [12, 31, 32] and how it exerts its activity [30]. We have investigated the subcellular localisation of PDCD4 in pancreatic cells maintained in normoxic and hypoxic conditions. Immunocytochemistry analysis of PDCD4 in ARIP cells showed that PDCD4 was highly expressed in the cytoplasm compared to the nucleus of the cells (Fig 4). In addition, weak expression of PDCD4 was observed in ARIP cells under conditions of serum starvation, hypoxia (12 hours) and normoxia (12 hours) although, strong PDCD4 expression was observed after 24 hours in both hypoxia and normoxia (Fig 4). In MIN6 cells PDCD4 was highly expressed in the cytoplasm compared to the nucleus under all conditions (Fig 4). In hypoxia (24 hours), PDCD4 was exclusively expressed in the cytoplasm (Fig 5). ICC findings also suggested that PDCD4 expression was localised to the cytoplasm, with no shuttling between the cytoplasm and the nucleus or vice versa under any of the conditions studied. To additionally confirm the accuracy and specificity of our western blot nuclear/cytoplasmic fractionation and the accuracy of our ICC staining protocols, the same analyses were performed for transcription factor HIF-1α (S2 and S3 Figs), which was demonstrated to localise specifically to the nucleus, with no detectable cytoplasmic staining.

The tumour suppressor PDCD4 has been linked to T1D [11], obesity linked diseases [37] and our previous studies have reported the role of *Pdcd4* in pancreatic islet neogenesis [12]. The exact role of PDCD4 during the process of apoptosis remains to be clarified, as some studies have identified it as being up-regulated [13] and others as down-regulated [38]. The current findings have indicated that PDCD4 is upregulated during the process of apoptosis in pancreatic cells exposed to periods of hypoxia.

In the present investigation we have attempted to define the mechanism of pancreatic cell death in response to hypoxia. Many studies have reported that pancreatic beta cell lines are sensitive to hypoxia because of the high rate of mitochondrial respiration required to support insulin secretion [5]. Our viability analyses confirmed that MIN6 cells are highly sensitive to hypoxia, since under hypoxic conditions a massive loss of cell population occurs, reaching approximately 70% in 24 hours following induction of apoptosis as well as necrosis (Fig 1).

The MTT results for ARIP cells showed no effect of hypoxia on growth, whereas HPI staining indicated that hypoxia triggered apoptosis following 24 hours of hypoxia. A potential explanation for the differing results obtained using the MTT and HPI assays in hypoxia might be that ARIP cells were in the early phase of apoptosis, as the early phase of apoptosis does not affect membrane permeability, or cause any alteration in the activity of mitochondria. Therefore, the MTT assay might be more effective for detecting the later stages of apoptosis [39, 40]. Thus MTT assay may therefore underestimate the levels of apoptosis if the apoptosis process is at an early stage. Also when comparing the viability of ARIP and MIN6 cells after exposure to hypoxic conditions we demonstrated detrimental effects on beta cell growth and viability, but this again was not observed in pancreatic ductal cells. These observations may have great relevance to the damaging processes experienced by β-cells during the procedure of islet isolation and transplantation [41, 42]. Hypoxia is an inevitable result of the devascularisation which occurs during islet isolation and purification; the subsequent transplantation into an environment without an oxygen supply ultimately results in a negative impact on beta-cell survival [43]. It is believed that after islet transplantation, revascularization of pancreatic islets may take several days [44] and β-cells will therefore experience hypoxia which may have a detrimental effect on β-cell function [43, 45, 46]. Revascularization and supply of sufficient oxygen to pancreatic islets can provide protection against hypoxia and ultimately can increase β-cells survival [46]. Our observations of β-cell viability support this need to maintain oxygen supply for beta cell survival, which could have a positive impact on islet transplantation procedures.

The possible involvement of PDCD4 in apoptosis has been reported in many studies and loss of its expression has been observed in many cancers, which makes PDCD4 a potential therapeutic target. Recently, it has been reported that hypoxia induces tumour cell resistance to apoptosis and results in decreased response to chemotherapy by up regulating miR-424 which in turn supresses the level of PDCD4 protein in A375 tumour cells [47]. Our results clearly indicated that hypoxia significantly induced the expression of PDCD4 in both ARIP and MIN6 cells. Pancreatic cell viability and PDCD4 western blotting analyses suggested that PDCD4 might play role in the induction of apoptosis in pancreatic cells. However, high expression of PDCD4 was observed in ductal and β-cells (ARIP and MIN6) following sustained hypoxia (24 hours) but was not detected in individual populations of apoptotic or necrotic cells. Thus, the present data suggests that there may be a link between high levels of PDCD4 expression and apoptosis or necrosis induced under hypoxic conditions in ARIP and MIN6 cells.

To attempt to answer the question of whether higher expression of PDCD4 in hypoxia induces apoptosis in pancreatic cells, we sought to determine if it is indeed only increased PDCD4 expression that induces the apoptosis or whether the subcellular localisation of PDCD4 also plays a role in its activity. How PDCD4 exerts its effects is still not clear, but it has been reported that in the cytoplasm, PDCD4 inhibits helicase activity of eIF4a as well as interfering with the interaction of eIF4a with eIF4G, ultimately resulting in an inhibitory effect on protein translation [29, 30]. Studies of the role of PDCD4 in apoptosis have also provided inconsistent findings; PDCD4 has a role in apoptosis in the case of lung [48] and breast cancer [49] however, no apoptotic effect has been found in other tissues [50, 51]. It is worth noting that in other cell types, PDCD4 has been implicated in a broad range of cellular processes, acting as a tumour suppressor through several alternative mechanistic pathways [52, 53]. Indeed even within pancreatic tissue we have previously shown that PDCD4 is linked to islet neogenesis in vivo, being expressed in both islet cells and pancreatic ductal cells [12]. Previous studies have suggested a potentially cell type specific role for PDCD4 [34]. It is certainly possible that PDCD4 plays more than one role within individual pancreatic cell types. Our data from immunocytochemistry analyses have shown that PDCD4 was mostly localised in the cytoplasm of pancreatic cell lines (ARIP and MIN6 cells) with no observed translocation or shuttling of PDCD4 between the cytoplasm and the nucleus, thereby complimenting the parallel western blotting findings.

**Fig 6:**
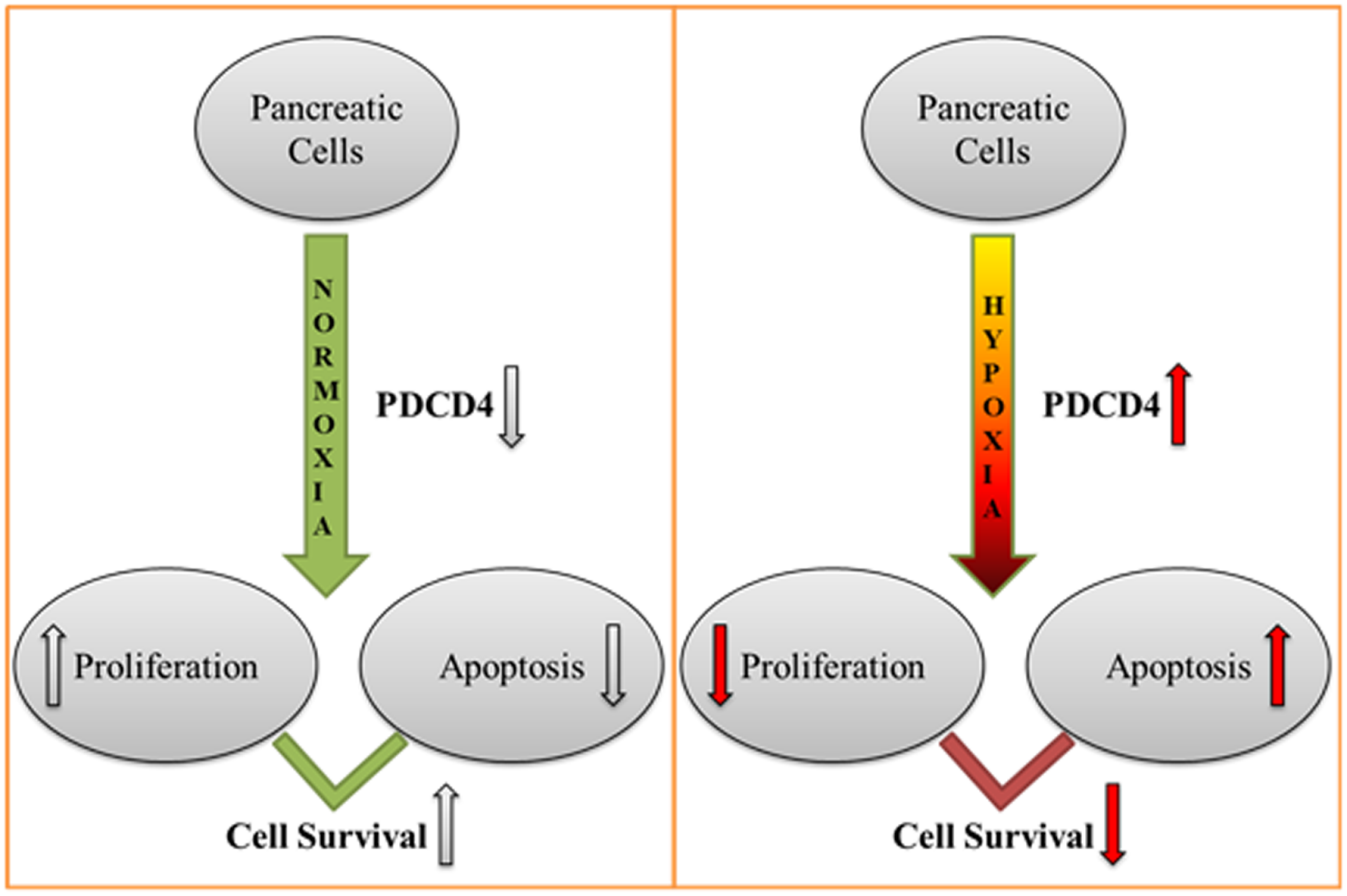
Schematic representation of the proposed effect of PDCD4 expression on cell survival in the pancreas. In this study, we have explored the role of hypoxia in controlling expression of PDCD4. Hypoxia (1% oxygen) induces the expression of PDCD4 in pancreatic cells such as MIN6 and ARIP cells, resulting in low proliferation and the triggering of apoptosis, whereas under normoxic conditions (21% oxygen) expression of PDCD4 stayed low resulting in cell proliferation and survival. The role of PDCD4 under hypoxic conditions may well be decided by the master regulator of oxygen homeostasis, HIF-1α.

Briefly, our data demonstrated that hypoxia induced expression of PDCD4 may be linked to the induction of apoptosis in both of the pancreatic cell lines studied. A summary of our proposed model is shown in Fig 6. These studies open up the exciting possibility that PDCD4 could provide a new potential molecular target in the therapy of T1D.

## Supporting information

S1 Fig Scanning electron microscopy of the surface morphology of mouse pancreatic beta cells (MIN6). MIN6 cells were fixed with 2.5% glutaraldehyde and coated with platinum coating (4nm). Cells were examined using Zeiss Sigma field emission gun SEM (Zeiss NTS). Images were captured at magnifications 1K (A-E), 10K (F-J) and 50K (K-O). Images were representative of six separate fields. At G0 (A, F & K), N12 (B, G & L) & N24 (C, H & M) cells grew in groups and formed tight intercellular (cell to cell) contacts. Under hypoxic conditions at 12 hours (D, I & N) cell numbers fell and blebbing of the plasma membrane was observed, indicating apoptosis (highlighted by arrows). Under hypoxic conditions at 24 hours (E, J & O), a very low population of cells was observed and plasma membrane pores indicative of necrosis were observed (highlighted by arrows). Scale bars for A-E = 10μm, F-J = 1μm and K-O = 200nm.
(TIF)

S2 Fig Subcellular localisation of HIF-1α in pancreatic ductal cells. ARIP cells were cultured under G0 (serum starvation), normoxic or hypoxic conditions for 12 or 24 hours. After each indicated incubation period, the cells were pelleted. Cytoplasmic and nuclear proteins were extracted and 10μg of cytoplasmic and nuclear cell extract were separated on a 10% SDS-PAGE. Proteins were western blotted using an antibody specific to HIF-1α. Panel A (I) represents HIF-1α (102kDa) protein expression in the cytoplasm (II) represents protein loading control β-Actin (42kDa). Panel B (I) represents HIF-1α (102kDa) protein expression in the nucleus (II) represents protein loading control lamin B1 (74kDa). Panel C illustrates densitometry analysis showing cytoplasmic HIF-1α relative to control β-Actin and nuclear HIF-1α relative to lamin B1. These results were reproduced in at least three separate experiments. Error bar values represent mean +/- standard error. HIF-1α was exclusively expressed in the nucleus under normoxic and hypoxic conditions. Expression of HIF-1α was significantly higher at H24 (***p<0.001) compared to G0. Also HIF-1α was significantly higher at H24 (**p<0.01) compared to N24.
(TIF)

S3 Fig Sub-cellular localisation and expression of HIF-1α in pancreatic ductal cells. ARIP cells were grown on glass cover slips in six well plates and fixed at specific time points i.e. at G0 (Serum starvation), N12 & N24 (Normoxic) and H12 & H24 (Hypoxic). Immunocytochemistry was performed using a specific antibody to HIF-1α and a FITC labelled secondary antibody. Coverslips with cells were mounted on glass slides with mounting medium containing DAPI which stains the nucleus of cells. Cells were analysed by confocal microscopy and images were captured at 65X magnification. Results are representative of three separate experiments and images were represented in six separate fields. HIF-1α was exclusively localized and expressed in the nucleus of ARIP cells. In addition, expression of HIF-1α was increased at H24 compared to G0.
(TIF)

## Abbreviations

HPI: Hoechst Propidium iodide
ICC: immunocytochemistry
PDCD4: Programmed Cell Death Gene 4
SEM: scanning electron microscopy
WB: western blotting
