# Supplementary figures and images for: The role of tumour suppressor PDCD4 in beta cell death in hypoxia
Source: PLoS One. 2017 Jul 27;12(7):e0181235. doi: 10.1371/journal.pone.0181235 (PMC5531437; doi:10.1371/journal.pone.0181235)

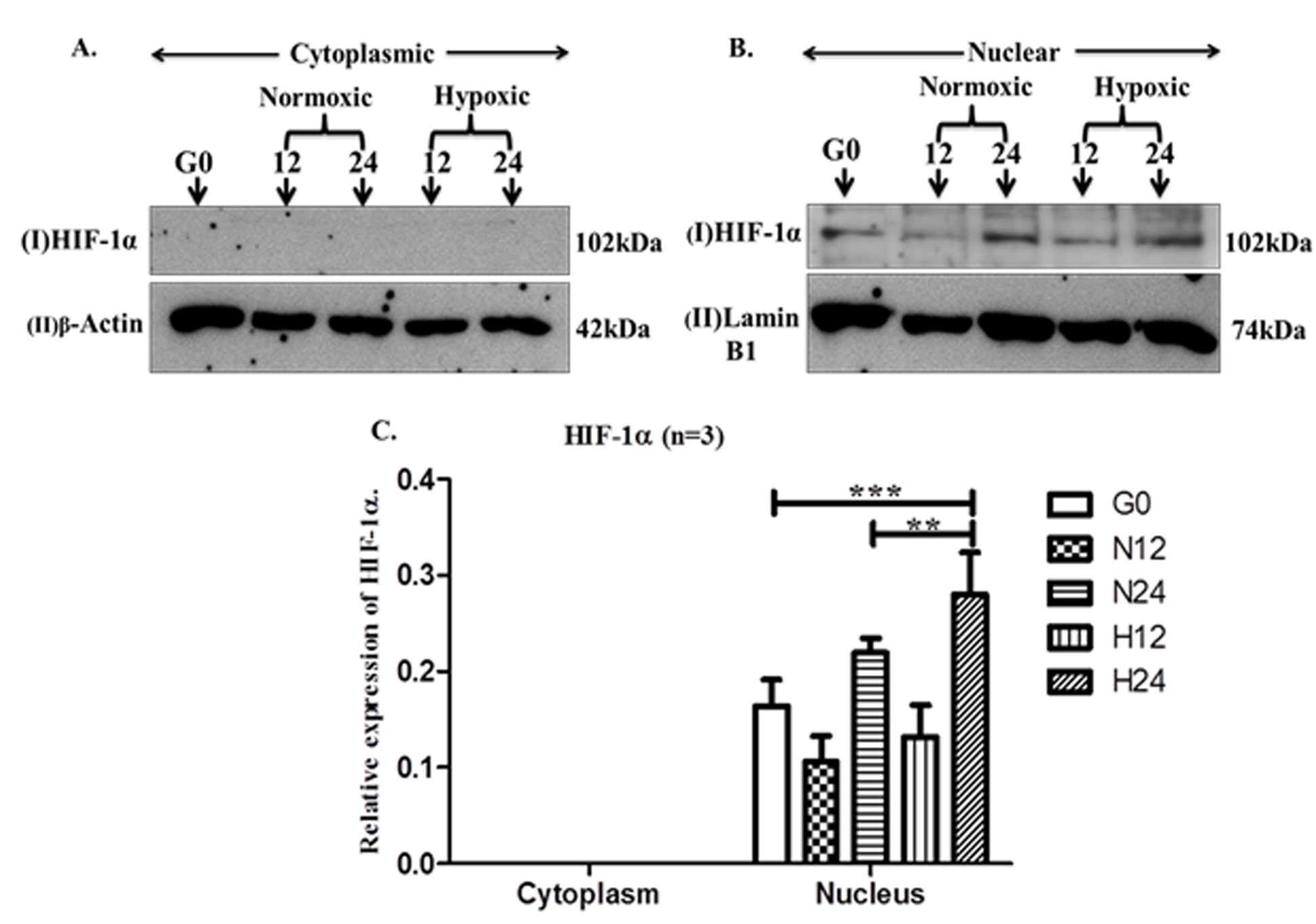

Supplement: S2 Fig — ARIP cells were cultured under G0 (serum starvation), normoxic or hypoxic conditions for 12 or 24 hours. After each indicated incubation period, the cells were pelleted. Cytoplasmic and nuclear proteins were extracted and 10μg of cytoplasmic and nuclear cell extract were separated on a 10% SDS-PAGE. Proteins were western blotted using an antibody specific to HIF-1α. Panel A (I) represents HIF-1α (102kDa) protein expression in the cytoplasm (II) represents protein loading control β-Actin (42kDa). Panel B (I) represents HIF-1α (102kDa) protein expression in the nucleus (II) represents protein loading control lamin B1 (74kDa). Panel C illustrates densitometry analysis showing cytoplasmic HIF-1α relative to control β-Actin and nuclear HIF-1α relative to lamin B1. These results were reproduced in at least three separate experiments. Error bar values represent mean +/- standard error. HIF-1α was exclusively expressed in the nucleus under normoxic and hypoxic conditions. Expression of HIF-1α was significantly higher at H24 (***p<0.001) compared to G0. Also HIF-1α was significantly higher at H24 (**p<0.01) compared to N24. (TIF) [file pone.0181235.s002.tif]

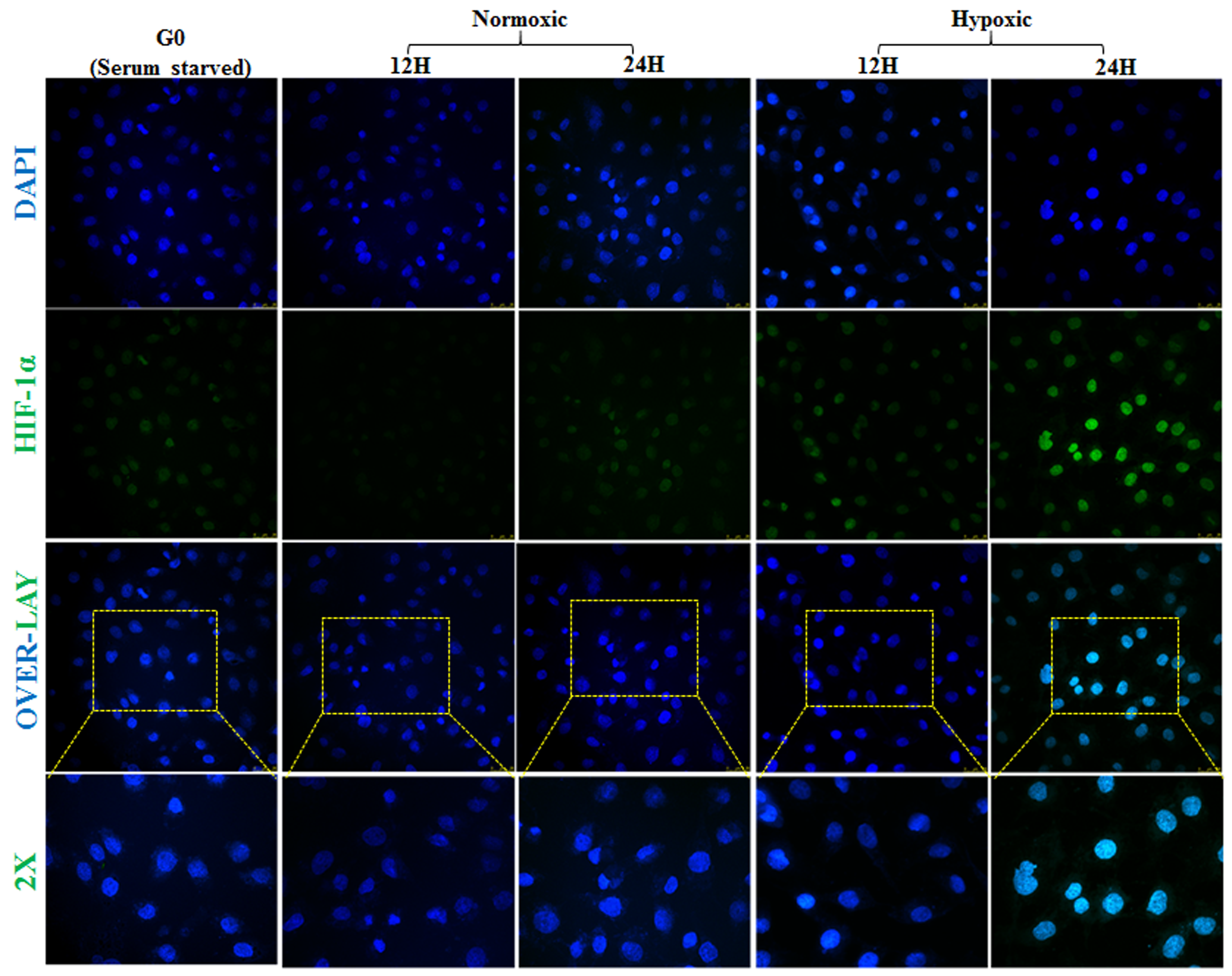

Supplement: S3 Fig — ARIP cells were grown on glass cover slips in six well plates and fixed at specific time points i.e. at G0 (Serum starvation), N12 & N24 (Normoxic) and H12 & H24 (Hypoxic). Immunocytochemistry was performed using a specific antibody to HIF-1α and a FITC labelled secondary antibody. Coverslips with cells were mounted on glass slides with mounting medium containing DAPI which stains the nucleus of cells. Cells were analysed by confocal microscopy and images were captured at 65X magnification. Results are representative of three separate experiments and images were represented in six separate fields. HIF-1α was exclusively localized and expressed in the nucleus of ARIP cells. In addition, expression of HIF-1α was increased at H24 compared to G0. (TIF) [file pone.0181235.s003.tif]
